# Democracy, green energy, trade, and environmental progress in South Asia: Advanced quantile regression perspective

**DOI:** 10.1016/j.heliyon.2023.e20488

**Published:** 2023-09-29

**Authors:** Tasnim Sultana, Md Shaddam Hossain, Liton Chandra Voumik, Asif Raihan

**Affiliations:** a Department of Economics, Noakhali Science and Technology University, Noakhali, 3814, Bangladesh; b Institute of Forestry and Environmental Sciences, University of Chittagong, Bangladesh

**Keywords:** Environmental degradation, Green energy, Democracy, STIRPAT model, Quantile regression, South Asia

## Abstract

Unquestionably, the industrial revolution of the twenty-first century contributes to global warming. Excessive amounts of carbon emissions into the atmosphere are responsible for global warming. Therefore, this research aims to assess the impact of GDP, green energy consumption, population, trade openness, and democracy on CO2 emissions in four selected South Asian countries from 1990 to 2019. This research also attempts to evaluate the EKC hypothesis in terms of economic growth (GDP2). The unit root of panel data and cointegration tests are executed in this study as a prelude to the regression analysis. Quantile regression for panel data, which (Powell, 2016) devised to deal with the fixed effect problem, is used in this study, and (Powell, 2016) empirical findings are the main focus. The estimated coefficient of GDP is positively significant, demonstrating that economic activity increases the burning of fossil fuels and upsurges atmospheric CO2 emissions. After attaining economic development, the reversed U-shaped EKC theory is valid for four selected South Asian countries. Economic development encourages these countries to use green technology, which helps mitigate CO2 emissions. The research, however, reveals that green energy is to blame for CO2 emissions. Burning biomass releases carbon dioxide that negatively impacts the quality of the environment. The study confirms that human activities are the leading contributor to environmental deterioration. Population growth has a worsening effect on the environment. The association between population and CO2 emissions is positively significant. The estimated coefficient of trade openness is positive, which increases CO2 emissions significantly. The estimated coefficient of democracy is quite negative. Therefore, this study suggests prioritizing democracy to reduce CO2 emissions. Citizens who live in democracies are better informed, more organized, and able to protest, all of which contribute to increased government responsiveness to environmental preservation. The results of the Wald test support the differential effects at various quantiles. The Dumitrescu-Hurlin (2012) panel causality tests are also used in this analysis to check causality between variables. Based on the findings, this research makes many policy suggestions for lowering carbon emissions.

## 1 Introduction

Climate catastrophe is one of the long-term difficulties confronting the modern world. Daily climate change is a matter of concern for the world population, environmental biodiversity, and economic sustainability. It is a crucial challenge for this generation because exacerbated climate change will put the next generation at risk. Therefore, the current generation should be aware of protecting the environment so that the next generation can grow up in a pollution-free, environment-friendly environment.

At the COP26 conference, there were great hopes that governments would make new commitments to reduce carbon emissions more effectively than ever [1]. Global leaders concurred that, in light of the severity of the climate problem, countries should submit more ambitious emission reduction plans to reduce global warming to 1.5° Celsius by 2030 [2,3]. The difficulties posed by climate change have intensified due to harmful greenhouse gases (GHGs). Fossil fuels have been used to meet energy needs, which increases carbon emissions and harms the environment [4]. Most *CO*_*2*_ emissions (about 90% of the total) come from burning fossil fuels [5,6]. It is essential to control the use of fossil fuels to reduce the ongoing release of carbon emissions [7]. Researchers and policymakers are becoming more worried about climate change because developed nations contribute much more carbon dioxide emissions than less developed nations [8]. Developing countries suffer more than they contribute to per capita *CO*_*2*_ emissions. No matter who is responsible, the concentration of GHG (greenhouse gas) emissions on the surface harms countries worldwide, those that have developed and those that are still developing [9]. [10] advised substantial adjustments to how fossil fuels are used since they pose a severe threat to the future. Investments in carbon-neutral technology are urgently needed if global warming is to be maintained well below 2° Celsius. Environment protection should not be solely the government's responsibility; it is high time to encourage people to make concerted efforts to eliminate environmental degradation [11]. It is essential to know how different variables affect *CO*_*2*_ emissions.

Carbon emissions are consistently rising due to the global economy, making it challenging to implement the Paris Agreement on climate change [12]. Most of the studies revealed not only a positive but also a significant influence of *GDP* on environmental degradation, including those of [[13], [14], [15], [16]]; and [17]. In South Asian nations [18], provided significant evidence in support of a positive correlation between *GDP* and *CO*_*2*_ emissions. Carbon emissions can be reduced in SAARC nations by adopting environmentally friendly production techniques [19]. [20] proposed a theory known as the EKC curve and investigated a dynamic reversed U-shaped linkage between economic growth and deterioration of the environment, particularly *CO*_*2*_ emissions. Some scholars firmly believe that economic growth (*GDP*^*2*^) is negatively associated with environmental contamination, which is well-matched with the reversed U-shaped EKC theory [21,22]; and [23]. Following the findings of a research project by Ref. [24]; the EKC hypothesis is accurate for MENA economies. Renewable energy is regarded as one of the finest sustainable development strategies in the developed world. Most researchers agree that increasing renewable energy is an excellent way to reduce carbon emissions. However, it is noteworthy that there is a similarity in most of the research that shows that the consumption of renewable energy helps to save the sustainability of the environment [[25], [26], [27], [28]]. However, biomass combustion causes environmental degradation by contributing to carbon emissions in G-7 nations [29]. The population is considered a direct source of *CO*_*2*_ emissions. Most studies have demonstrated that population growth enhances environmental degradation [[30], [31], [32]]. The research by Ref. [33] found the opposite result; they analyzed a negative and insignificant influence of population growth on ecological footprints because the population in developed economies uses green technology. After all, they are educated and concerned about the environment. The opening of trade has impacted carbon emissions positively, as validated by a wide range of previous studies, including those of [34,35]; and [36]. According to the research of [37]; increased trade openness leads to a dramatic increase in *CO*_*2*_ emissions, degrading environmental quality [38]. researched the association between carbon dioxide emissions and trade openness in SAARC countries. They used suitable panel data techniques and found that trade openness negatively affects *CO*_*2*_ emissions. Several research findings found the opposite results, showing that trade openness encourages nations to use resources more effectively, which benefits the environment, as confirmed by Refs. [39,40]. When a democracy strictly adheres to environmental laws and regulations and takes part in global environmental agreements, it is claimed that democracy has a substantial impact on how the economy develops [41]. In democracies, the whole population can obtain information about environmental quality. They can voice their opinions and pressure their governments to improve the environment [42]. If a nation has already attained a certain income level, democracy can lower *CO*_*2*_ emissions [43]. Also, Zandi et al. (2019), [12]; and Jahangir et al. (2020) all agree that democratic government awakened people to reduce emissions of *CO*_*2*_ [44]. found a progressive relationship between democracy and environmental commitment, with democratic-based nations showing more significant concern for the environment than non-democratic nations. Midlarasky (1998) found a reverse influence between democracy and the quality of the environment, whereby environmental performance is worsened by democracy.

South Asia is a subregion of Asia consisting of Bangladesh, Bhutan, India, Nepal, Pakistan, and Sri Lanka. Afghanistan and the Maldives are also regarded as parts of South Asia. Since the 1990s, trade liberalization has also gained much attention in South Asian countries because trade liberalization will facilitate regional trade, especially in the export sector. Due to trade liberalization, developed countries extensively invest in the industrial sectors of third-world countries. There are many reasons developed countries invest in third-world countries: the environmental regulations are pretty lax, industrial economies of scale can be easily achieved, and production costs are reduced because skilled labor is readily available [45]. While trade liberalization has positive aspects, it is also associated with environmental degradation. Trade openness increases the income of the exporting country. It stimulates economic activity, causing governments to engage in more non-environmentally friendly activities to increase industrial output, which causes rising carbon emissions [46]. In the SAARC (South Asian Association for Regional Cooperation) region, India and Pakistan are the top two producers of carbon dioxide [47]. Most South Asian countries account for the world's most populous nations [48]. Around twenty percent of the world's population lives in the South Asian region [49]. After the end of autocratic rule, South Asia became the most significant region with a democracy [50]. South Asian governments face continuous obstacles to achieving Sustainable Development Goals [51].

South Asia is known as the most populous region in the world. The region is highly vulnerable to climate change due to excessive carbon build-up. That is attributed to countries' significant economic growth, trade liberalization, and population growth. Since democratic governments exist in South Asian countries, knowing their role in dealing with such situations becomes essential. In this context, this research aims to assess the influence of *GDP, GDP*^*2*^, *green* energy consumption, population, democracy, and trade openness on *CO*_*2*_ emissions of four selected South Asian countries. These selected countries are Bangladesh, India, Pakistan, and Sri Lanka. We have selected these four countries for our study as data for other South Asian countries is unavailable. Although there is a weak democracy presence in Pakistan, according to the International Country Risk Guide (ICRG), Pakistan's average score (2.36) on the democracy index is comparatively lower than Bangladesh and India, two other South Asian countries, from 1984 to 2017. Pakistan's political institutions are still in the early phases of development due to more than 35 years of military dictatorship, although they are gradually improving [52]. Hence, Pakistan is also prioritized in the study because the impact of democracy on the environment is noticed. Researchers and policymakers worldwide are interested in this study area because they want to know if these variables are linked to carbon emissions.

As a result, the study adds several valuable contributions that enhance the literature. Firstly, the novelty of our research is that there has not been much study of democracy in these regions. Previous empirical research yielded mixed findings regarding the connection between democracy and the environment. According to the scenario mentioned earlier, the research is anticipated to disclose the links between carbon emissions and democracy, which will be more pertinent for determining environmental policy. Secondly, in the study of democracy, most of the research used data gathered from the Household Freedom Index. The study differs from existing literature because we use data from the v-dem (variety of democracy) Electoral Democracy Index, where electoral democracy is viewed as a critical component of representative democracy [53]. Compared to earlier studies, it becomes clear that they employed data on electoral democracy from the V-dem index for a different sample of countries [54]; Adams & Acheampong; and [55]. The democratic electoral principle ensures that government politicians are accountable to the public [56]. Here, political organizations are allowed to operate freely, and elections are fair and free of systematic irregularities. In between elections, there is freedom of speech that can present opposing viewpoints on important political issues [56]. Thirdly, the study is the first work for South Asia that shows the impact of different variables on carbon emissions using [57] quantile regression, which gives an unconditional quantile treatment effect and permits using many control variables without biasing the results [58,59]. used [57] quantile regression method to investigate the environmental compatibility of Latin America. The study used the STIRPAT model and incorporated democracy and trade openness as proxies for Technology (T). The study uses the STIRPAT framework to understand the factors influencing environmental degradation comprehensively. The study incorporated democracy into the STIRPAT model, which also helps to evaluate the EKC hypothesis. Therefore, the empirical results will explain whether the practice of healthy democracy helps in the formation of EKC by increasing economic growth. In addition, the research highlights the policy gaps that need to be filled to make the world less polluted and more sustainable to deal with climate disasters. The theoretical and empirical estimates confirm that the gross domestic product (*GDP*), *green* energy consumption, population, trade openness, and democracy crucially and significantly affect carbon dioxide emissions in South Asian countries.

The study employs descriptive statistics and the Jarque-Bera test to see the data's normality. Next, the study checks the unit root problem using first and second-generation panel unit root tests. Next, the research employs the cointegration test to determine whether the variables are linked in the long run. After confirming that data sets are not normally distributed, the study adopts the quantile regression method proposed by Ref. [57]. The study uses the Wald test to see the significant asymmetric impacts of different variables on carbon emissions at different quantiles. The [60] panel causality test is also executed in the study, which looks for causal associations between variables.

The paper is organized in the following sequence: Section 2 reviews the most relevant literature in the field to support our conclusion and demonstrates the existing gap. Section 3 constructs the method used, data sources, variables, and estimation models; Section 4 reveals the estimation results and the discussions accordingly; and the last section of the paper outlines the conclusions and offers policy implications for the four selected South Asian countries.

## 2 Review of literature

[61] performed a study covering selected next-11 economies from 1990 to 2019 to see the impact of *GDP*, *GDP2*, population, and renewable energy consumption on carbon emissions. Their empirical findings from the MM-QR (method of moment quantile regression) approach suggested that *GDP* has a positive and *GDP2* has a negative association with carbon emissions, confirming the EKC theory's validity. Their study also confirmed that population growth and renewable energy consumption impede the release of carbon into the atmosphere [54]. assessed the linkage between *GDP*, population, renewable energy consumption, and carbon emissions in BRICS economies from 1990 to 2019. Their study revealed that *GDP* growth and population expansion significantly promoted atmospheric carbon dioxide emissions. Renewable energy consumptions and democracy play a positive role in preserving environmental sustainability in BRICS [62]. examines the factors that determine GHG emissions in South Asian nations, indicating that GDP has a substantial long-term impact while fossil fuels are the primary source of atmospheric emissions. Their analysis highlights the crucial role that nuclear and renewable energy play in reducing pollution, emphasizing the necessity for comprehensive green energy regulations [63]. studied the nexus between per capita *GDP* and environmental deterioration during the period 1990–2019 in the context of Asian countries that are developing. Based on autoregressive distributed lag (ARDL) and pooled mean group (PMG) estimation, inverse U-pattern EKC is not validated for these countries. To investigate population and *GDP*'s influence on ecological footprints [33], used data ranging from 1980 to 2015 in 12 selected advanced countries. Following the CS-ARDL findings, economic activity increases the ecological footprint significantly, while population growth decreases it insignificantly.

Another investigation is conducted on the impact of trade openness, economic growth, and renewable energy penetration on carbon dioxide emissions in Pakistan by Ref. [64] over the period 1990–2017 using F-tests, unit root tests, and cointegration tests. The authors concluded that trade openness and economic growth are to blame for worsening environmental conditions since they lead to higher *CO2* emissions. Also, they found that renewable energy penetration impedes ecological degradation [18]. examined the existence of the hypothesis of EKC in five selected South Asian nations. The study, which employed the autoregressive distributed lag (ARDL) approach, provided long-term support for the EKC theory in Pakistan and Sri Lanka and short-term support for the theory in Bangladesh. Using panel data for 1990–2018 [65], investigated how *GDP* and renewable energy consumption in selected next-11 countries affect ecological footprints. The quantile regression approach asserts that ecological footprints are positively associated with renewable energy consumption and per capita *GDP*.

[66] consider the impact of renewable energy, nuclear power, and R&D on the environmental Kuznets curve (EKC) hypothesis in the European Union (EU). The authors analyze panel data to learn how *CO2* emissions, economic growth, and renewable energy sources are linked. The study offers new evidence of the capabilities of renewable energy to reduce carbon emissions and boost long-term economic growth in the EU. The results can help policymakers craft more efficient plans to advance regional environmental and economic objectives. Using GMM calculations for annual cross-sectional data from 2000 to 2017, Naseem & Ji (2021) investigated how SAARC countries' renewable energy use and economic growth affected *CO2* emissions. They suggested that using more renewable energy significantly decreases *CO2* emissions, while *GDP* growth greatly increases atmospheric carbon dioxide [67]. investigate the intermittent impacts of renewable energy, non-renewable energy, natural resources, human capital, and globalization on the ecological footprint in developing countries. Their research offers valuable evidence of the complex relationship between these factors, shedding light on these nations' sustainability challenges. This study contributes to understanding the intricate interplay between renewable energy, resources, human capital, and globalization in shaping environmental impacts [68]. explore the relationship between institutional quality, renewable energy consumption, and *CO2* emissions in developing countries. Their study employs various econometric techniques to provide empirical evidence on the moderating role of institutional quality in the energy-environment nexus. This research contributes to understanding policy implications for sustainable development and offers insights for mitigating *CO2* emissions in developing economies.

[31] used GLM (Generalized Linear Model), GMM, and robust least squares to examine the role of population growth and renewable energy consumption on environmental sustainability in the United States from 1971 to 2016. The research exposed a positive nexus between population growth and carbon dioxide emissions, while renewable energy consumption has a negative association with carbon dioxide emissions. Over the period 1984–2015 [35], investigated how economic growth and trade openness affect the quality of the environment in South Asia. The findings revealed that a reversed U-shaped association exists between economic growth and environmental degradation, and South Asia's ecological conditions would worsen as trade openness increased [69]. analyzed to investigate the impact of democracy, renewable energy consumption, and economic growth on ecological footprint in the seven most developed G-7 nations over the period 1985–2017 using the econometric techniques of the second generation (CUP-FM and CUP-BC). They found that democracy and renewable energy enhance ecological sustainability, but *GDP* growth degrades environmental quality by aggravating the ecological footprint [70]. conducted a study using a panel quantile regression approach in their research for West African nations. Their empirical findings suggested that *GDP* affected *CO2* emissions negatively, while *GDP*^*2*^ affected them positively. These negative and positive values confirmed the U-shaped association between economic growth and carbon emissions [25]. examined five SAARC countries for panel data covering the years between 1990 and 2013 and investigated how using renewable energy affected the overall environmental quality standards. According to the study's findings, more significant usage of renewable sources can contribute to reducing carbon emissions.

Using FMOLS and DOLS [71], conducted a study in 11 nations in South Asia during the period 1960–2014. The result proved that the association between population size and carbon emissions is positively significant. He also found that the EKC is invalid for South Asia because the link between economic growth and carbon emissions is U-shaped [72]. investigated the nexus that exists between carbon emissions and democracy in the top 6 ASEAN countries during the period 1995–2017. Their research used FMOLS and DOLS estimation techniques and demonstrated that democracy helps minimize *CO2* emissions [73]. investigated how democracy affected PM2.5 in G-20 nations. By utilizing panel quantile regression models, the authors discovered that democracy vitiates environmental quality. If a country has significant corruption, democratic governments have failed to reduce carbon emissions [55].

After evaluating the primary research, we observe that various studies in South Asia have examined the association between *GDP*, renewable energy use, and environmental pollution; however, only some studies have relied on sufficient data. The studies above reveal that most attempts to determine the primary reasons for ecological contamination have yielded conflicting findings. The variables employed in these existing studies did not capture all relevant factors that degrade the environment in the South Asian region. The study used the STIRPAT model, which can help effectively measure the influence of factors such as population growth, affluence, and technological advancement on *CO*_*2*_ emissions. From the existing literature, it can be said that not much work has been done on this model and these variables in South Asian countries. In comparison with previous studies, we found that the existing literature for South Asia did not use the Powell quantile regression method to identify the reason for environmental degradation. Simultaneously, the study incorporated various econometric techniques, such as the stationarity test, cointegration test, OLS pooled, fixed effect, random effect, panel quantile regression approach, wald test, and causality test, to provide a clear picture of the factors influencing carbon emissions via a rhythmic analysis.

## 3 The model and econometric methodology

### 3.1 Data, variables, and sources

The main motive for the research is to determine whether democracy and renewable energy consumption matter in improving environmental quality. The study also attempts to assess the influence of *GDP*, *GDP*^*2*^, renewable energy consumption, population, and trade openness on *CO*_*2*_ emissions in selected South Asian countries between 1990 and 2019. The research investigation uses secondary data gathered from various external sources. The study has listed all the variables information in Table 1 for the readers' convenience.

**Table 1:** Data classification and units of measurement.

Variable name	Indicator	Units of Measurement	Source
Carbon dioxide emissions	*lnCO*_*2*_	CO_2_ emissions (kilotons)	WDI
Gross Domestic Product	*lnGDP*	GDP (constant 2015 US$)	WDI
Square of GDP (Gross Domestic Product)	*lnGDP*^*2*^	Square of GDP (constant 2015 US$)	WDI
Renewable/Green Energy Consumption	*lnREN*	Renewable energy consumption (% of total final energy consumption)	WDI
Population	*lnPOP*	Population Size (population, total)	WDI
Trade Openness	*lnTOP*	The ratio of exports plus imports to GDP	WDI
Democracy	*DEMO*	Electoral democracy data	Varieties of Democracy (V-dem) index

### 3.2 Theoretical framework and STIRPAT model

The study's dependent and independent variables recommend employing an upgraded version of the IPAT model, the well-known STIRPAT model [74]. insists that the IPAT model is widely employed to examine how anthropogenic activity affects environmental quality [75]. appeared to be the first to develop the IPAT framework. These IPAT affiliations are essential and relevant to environmental concerns [76]. Moreover, it is a mathematical identity that specifies precisely how factors relate to their impacts [77]. To express the IPAT equation mathematically, it can be stated as following Equation (1.1):

(1.1) I=P.A.T

in this case, based on the IPAT framework, “I" stands for the impacts of the environment, “P" for population size, “A" for affluence, and “T" for technology. In the study, the total population is a proxy for the population, the *GDP* is a proxy for affluence, and the consumption of renewable energy is a proxy for technology. *CO*_*2*_ emissions are used to assess environmental conditions in this case. Even though the IPAT framework can quickly find the most critical environmental change factors, it has been found to have some problems. Other factors, human behavior, and decisions impacting environmental quality have yet to be considered as determining factors in the IPAT model [78,79]. [80] are the ones that came up with the idea for the model known as the STIRPAT model, which is known as an improved version of the IPAT model and addresses all of the IPAT model's drawbacks. The stochastic (ST) impacts (I) through regression (R) on population (P), affluence (A), and technology (T) are abbreviated as the STIRPAT model. The STIRPAT model introduces the idea of ecological elasticity (EE), which allows for a more precise identification of the sensitivities of environmental impacts to the factors causing those [77]. The STIRPAT models combine various technological elements, including using democracy, *green energy*, human capital, industrialization, technological innovation, and trade openness [16]; Usman et al., 2022a; [43,81,82]. In the extended model, we incorporate democracy and trade openness as a proxy for technology to examine how these factors influence the condition of the environment. We also include the square of *GDP* in the model to determine whether the EKC hypothesis is valid in the four selected South Asian countries. Kuznets was the first person to propose the concept of a relationship that is an “inverted U-pattern” between economic development and the inequality of income [83]. The study by Ref. [20] is best known, which identified a backward U-shaped association between *CO*_*2*_ emissions and income, commonly referred to as the EKC hypothesis. The EKC hypothesis proposes a dynamic process of evolution in which emission levels initially increase as income in an economy increases, income reaches a peak point (known as a turning point), and emissions fall after a certain income level is achieved.

Therefore, the extended STIRPAT model used in the analysis takes the following Equation (1.2):

$$\ln\;C{O_{2}}_{it}=\alpha_{0}+\alpha_{1}\;\ln\;GDP_{it}+\alpha_{2}\;\ln\;GDP_{it}^{2}+\alpha_{3}\;\ln\;REN_{it}+\alpha_{4}\;\ln\;POP_{it}$$

(1.2) $$+\alpha_{5}\;\ln\;TOP_{it}+\alpha_{6}DEMO_{it}+\varepsilon_{it}$$

We do not use logarithms for democracy because the data for democracy is already in percentage form. Here $\alpha_{0}$ are the intercept term and $\alpha_{1},\alpha_{2},\alpha_{3},\alpha_{4},\alpha_{5},\alpha_{6}$ the slope coefficients. Where $CO_{2}$ represents carbon emissions determined by gross domestic product (GDP), economic growth over time $\left(GDP^{2}\right)$, renewable/green energy (REN) consumption, population (POP) growth, trade openness (TOP), democracy, (DEMO) and a stochastic or random error term $\left(\varepsilon_{it}\right)$.

### 3.3 Econometric methodology

A balanced data set from 1990 to 2019 from four selected South Asian countries make up our sample. The stationarity and cointegration tests are indispensable when employing panel data with econometrics. There are two types of methods to estimate static panel data. One method we use is a pooled OLS regression model. The other estimation method is the fixed-effect or random-effect model. We must perform fixed and random effects in a static panel analysis, which are more appropriate if N > T [84]. The study uses the Hausman (1978) test to decide whether a fixed effect model or a random effect model is better for the analysis—the Hausman test aids in selecting the best model (Leitao, 2021). The study applies the quantile regression method because the Jarque-Bera test reveals that the data for most variables are not normally distributed. The key findings are based on [57] quantile regression method because the estimated simultaneous quantile regression approach does not correct the endogeneity issue with fixed effects variables in panel quantile regressions. However, the study also employs the Wald test to determine whether the different quantiles' asymmetric effect is statistically significant. Lastly, we do a causality test, which helps us make specific policies that reduce carbon emissions more effectively.

### 3.4 Panel unit root test

To test for stationarity, we perform the widely used unit root test. The famous first-generation unit root tests Im, Pesaran, and Shin [85] and Fisher-type [86] are used in our study. If a cross-sectional dependence problem exists, we must use the second-generation unit root tests, mainly the cross-sectionally augmented Dickey-Fuller (CADF) and cross-sectionally augmented Im, Pesaran, and Shin (CIPS) proposed by Ref. [87]. CIPS and CADF generate more reliable and trustworthy results when assessing the stationarity of variables.

### 3.5 Cointegration test

Researchers execute cointegration tests to check the validity of the long-term relationship between the variables. Several methods for testing panel cointegration have been used in our study, developed by Refs. [88,89]; and [90]. These panel cointegration tests are available with different advantages.

### 3.6 Panel quantile regression approach

The OLS regression does not distinguish between different cross-sectional units and only permits determining the conditional mean [43]. When the fundamental tenet of linear regression is broken, the quantile regression method is applied. To circumvent the limitations posed by the pooled OLS method [91], came up with the quantile regression approach. It can give distributional information about points besides the conditional mean. It provides a comprehensive picture of how an independent variable affects a dependent variable at different quantile levels. The study estimates the simultaneous quantile regression approach that [92,93] proposed to determine whether the effects of our variables are accurate and verify the expected sign. For the Quantile regression model, the following Equation (1.3) is:

(1.3) $$\begin{array}{l} Q_{\ln\;C{O_{2}}_{it}}\left(\tau /\alpha_{0},{\varepsilon_{i}}_{t},x_{it}\right)=\alpha_{0}+{\varepsilon_{i}}_{t}+{\alpha_{1}}_{\tau }\;\ln\;GDP_{it}+{\alpha_{2}}_{\tau }\;\ln\;GDP_{it}^{2}+{\alpha_{3}}_{\tau }\;\ln\;REN_{it}+\\ {\alpha_{4}}_{\tau }\;\ln\;POP_{it}+{\alpha_{5}}_{\tau }\;\ln\;TOP_{it}+{\alpha_{6}}_{\tau }DEMO_{it} \end{array}$$

where τ is a quantile in (0, 1) and $Q_{\ln\;C{O_{2}}_{it}}\left(\tau /\alpha_{0},{\varepsilon_{i}}_{t},x_{it}\right)$ is the τ−th conditional quantile function, and x_it_ means all the independent variables.

To fit the quantile regression for the panel data (QRPD) with a fixed effect that is nonadditive, Powell (2015) introduced a new econometric model, which was further developed in 2016 [94]. Since the estimated simultaneous quantile regression approach does not resolve the fixed effect problem [57], method is used here. This methodology corrects the endogeneity issue with fixed effects variables in panel quantile regressions [59]. The model of [57] quantile regression is as following Equation (1.4):

(1.4) $$\ln\;C{O_{2}}_{it}=\Sigma_{j=1}^{6}\;\ln\;E_{it}\alpha_{j}\left(\mu_{it}\right)$$

Where $\ln\;C{O_{2}}_{it}$ is, the endogenous variable, $\ln\;E_{it}$ is the combination of explanatory variables, the parameters of the values are indicated by $\alpha_{j}$ and $\mu_{it}$ demonstrate several disturbance terms.

Using OLS pooled regression offers a comprehensive overview of the relationships between democracy, *green* energy, *GDP*, population, trade, and environmental progress in South Asia by analyzing the pooled data in the South Asia region. This approach allows for a broader understanding of the general patterns and trends across the entire dataset. OLS fixed regression, on the other hand, offers insights into the specific effects of each independent variable on the dependent variable by controlling for country-specific characteristics. By accounting for individual country heterogeneity, this technique enables a more nuanced examination of the unique impact of democracy, *green* energy, *GDP*, population, trade, and environmental factors within every South Asia. The application of quantile regression provides valuable information on the conditional effects of independent variables at different points in the distribution of the dependent variable. By capturing the variations across different quantiles, this technique helps identify potential nonlinear relationships and assesses how the independent variables influence the outcome variable under other conditions. Combining these techniques offers a robust and multifaceted analysis of South Asia's complex dynamics between dependent and independent variables. It provides a more comprehensive understanding of the associations, accounting for various factors and allowing for a nuanced examination of the effects across different quantiles and country-specific characteristics.

### 3.7 Wald test

The study uses the Wald test to determine whether the coefficient values at different quantiles differ significantly.

### 3.8 Causality test

Regression analysis cannot establish a causal connection between the variables. The causality test helps identify the specific policy implications for tackling carbon emissions. Granger developed a test to evaluate the causal relationships between the variables [95]. It has some limitations; if a cross-sectional dependency problem exists in panel data, it does not apply. In this study, we employ an advantage causality test known as the D-H panel causality test, which was initially developed by Ref. [60]. The D-H panel causality test considers cross-sectional dependence problems in determining whether or not two variables are related.

## 4 Analysis of estimated outcomes

The results of descriptive statistics, which highlight the essential features of the variables, are displayed in Table 2. All variables in our analysis have uniform mean statistics. The value of *lnGDP*^*2*^ is the minimum, and *lnGDP* is the maximum, among other variables. The skewness of the value for *lnCO*_*2*_ is 0.368, so we can readily state that the right tails of *lnCO*_*2*_ have positive skewness. Here, the kurtosis value 1.991 for *lnCO*_*2*_ means platykurtic or negative kurtosis. The Jarque-Bera test outcomes indicate that most sample data do not have a normal distribution. For *lnCO*_*2*_, the Jarque-Bera test value is 7.8, which is significant at the 5% significance level. The study agrees with the alternative hypothesis and concludes that the distribution of *lnCO*_*2*_ is not normal.

**Table 2:** Descriptive Statistics (after logarithm).

Variables	*lnCO*_*2*_	*lnGDP*	*lnGDP*^*2*^	*lnREN*	*lnPOP*	*lnTOP*	*DEMO*
Observation	120	120	120	120	120	120	120
Mean	11.333	25.891	1.554	3.915	18.801	3.512	.497
Std. Dev	1.822	1.251	.398	.241	1.448	.472	.149
Minimum	8.253	23.802	.004	3.209	16.668	2.161	.204
Maximum	14.714	28.619	2.126	4.358	21.035	4.083	.763
Skewness	.368	.498	−1.461	−.575	.027	−1.156	.274
kurtosis	1.991	2.391	5.926	2.987	1.987	3.413	2.264
Jarque-Bera test	7.8**	6.8**	81.9***	6.60**	5.14*	27.5***	.4.20

Notes: ***p < .01, **p < .05, *p < .1.

Economists working with panel data must first test for CSD. The outcomes of analysis for CSD are shown in Table 3, where it is apparent that all panel data is affected by CSD (as evidenced by the rejection of the no-cross-sectional-dependence null hypothesis) because Pesaran scaled LM statistics have a p-value of more than 10%. Therefore, due to their economic and political similarities, democracy, renewable energy, *GDP*, population, trade, and environment exhibit CSD. Only one test shows that the result is insignificant.

**Table 3:** Outcomes of CSD analysis.

Tests	Test statistics	*P*-value
Pesaran CD test	14.026***	0.001
Pesaran scaled LM	4.42	0.115
Friedman test	98.256***	0.000
Breusch-Pagan LM test	124.021***	0.001

***shows 1% significant, ** shows 5% significant and * shows 10% significant.

Table 4 shows the results of the first-generation and second-generation unit root tests. These tests reveal that some variables remain stationary at the level denoted by the symbol I (0). However, other variables become stationary after just one difference, as shown by the symbol I (1). Table 4 shows that *lnGDP*^*2*^ and *lnPOP* are significant and stationary at a level, whereas *lnCO*_*2*_, *lnGDP*, *lnGDP*^*2*^, *lnREN*, *lnPOP*, *lnTOP*, and *DEMO* are stationary at the first difference. After taking the first difference, or I (1), the results of the IPS for all estimated variables are statistically significant with a 1% significance level. As a result, we accept the alternative hypothesis, implying that the panel data are stationary. The p-values of *lnCO*_*2*_ in Fisher ADF are more extensive than 10% at the I (0) level. So, the variables' first-order difference is further computed, and the first difference's p-values findings are significant, at least at the 10% level. The provided findings of IPS, ADF, CIPS, and CADF show that every variable produces a stationary result at the first difference.

**Table 4:** Results of Panel Unit Root test.

Variable	IPS		ADF		CIPS		CADF	
	I (0)	I (1)	I (0)	I (1)	I (0)	I (1)	I (0)	I (1)
*lnCO*_*2*_	−0.7481	−3.50***	10.4077	… …	−2.22*	−5.3***	−1.652	−3.06***
*lnGDP*	1.1894	−2.95***	6.6429	25.77***	−1.527	−3.9***	−1.320	−2.92***
*lnGDP*^*2*^	−2.69***	−6.50***	25.45***	68.27***	… ….	… ….	… …	… ….
*lnREN*	1.2020	−2.74***	6.2180	23.42***	−1.280	−4.7***	−1.079	−2.63**
*lnPOP*	−2.316**	−6.40***	20.25***	73.08***	0.302	−1.782	−3.21***	−4.88***
*lnTOP*	1.1927	−4.13***	4.2752	37.63***	−3.17***	−5.3***	−2.44**	−4.26***
*DEMO*	0.7270	−3.83***	11.2805	37.78***	−0.836	−3.8***	−1.488	−3.11***

Notes: ***p < .01, **p < .05, *p < .1.

After checking the unit root, long-run cointegration determination is needed as an estimation technique because the data set contains CSD issues. In this work, various testing procedures for panel cointegration were applied, which are displayed in Table 5. The cointegration results from Refs. [88,89,96] show that the null hypothesis of no cointegrating is not accepted, so we can declare that our variables are cointegrated in the long run. Again, the alternative hypothesis for the [90] test suggests that some panels have cointegrated variables in the long run. Cointegration is confirmed by the Kao, Pedroni, and Westerlund results, making it possible to estimate the model for a long-term connection.

**Table 5:** Results of Cointegration tests.

[88] test for cointegration	Statistic	p-value
Modified Dickey-Fuller t	−1.6426	0.0502*
Dickey-Fuller t	−1.7065	0.0440**
Augmented Dickey-Fuller t	−2.0079	0.0223**
Unadjusted modified Dickey-Fuller t	−1.6647	0.0480**
Unadjusted Dickey-Fuller t	−1.7149	0.0432**
[89,96] **test for cointegration**		
Modified Phillips-Perron t	0.9408	0.0734*
Phillips-Perron t	−2.7548	0.0029***
Augmented Dickey-Fuller t	−2.3233	0.0101**
H_0_: No Cointegration; H_a_: All panels are cointegrated		
[90] **test for cointegration**		
Variance ratio	−0.9259	0.0772*
H_0_: No Cointegration; H_a_: Some panels are cointegrated		

Notes: ***p < .01, **p < .05, *p < .1.

Table 6 shows that all regression coefficients in OLS pooled are highly statistically significant and consistent with prior expectations. The coefficient of the *lnGDP* is positive and significant at the 1% significance level. Carbon emissions grow by 1.21% for every 1% increase in *GDP*. However, at a 1% significance level, the estimates of *lnGDP*^*2*^ have a negative impact on carbon emissions, which proves that the EKC framework with the reverse U-shape is appropriate in the context of our investigation. The slope coefficient of *lnGDP*^*2*^ is −0.216, which indicates that the growth of carbon emissions would fall by 0.216% if *GDP* growth were further increased by 1%. With a 1% significance level, the estimated renewable energy consumption coefficient (*lnREN*) is positive. In four selected South Asian countries, there is a 1.09% rise in *CO*_*2*_ emissions for every 1% increase in renewable energy consumption. It is estimated that the population growth slope coefficient is significantly positive. According to the findings, carbon emissions will increase by about 0.382% for every 1% increase in population growth. A positive association exists between increased trade openness and increased *CO*_*2*_ emissions, which is significant at the 1% significance level. In particular, every increase of 1% in trade openness leads to a 0.47% rise in carbon emissions. Democracy is a significant variable in explaining *CO*_*2*_ emissions. With a 10% significance level, the association between democracy and carbon emissions is negative. Carbon emissions are reduced by 0.406% for every 1% rise in democracy (*DEMO*).

**Table 6:** Results of Pooled OLS, Fixed Effect, Random Effect Regression, and Hausman specification test.

Results of Pooled OLS, Fixed Effect, and Random Effects Regression			
*Variables*	(1)	(2)	(3)
*lnCO*_*2*_	OLS-Pooled	OLS-Fixed	OLS-Random
*lnGDP*	1.212***(0.083)	0.603***(0.067)	1.212***(0.083)
*lnGDP*^*2*^	−0.216***(0.055)	−0.013 (0.028)	−0.216***(0.055)
*lnREN*	1.097***(0.184)	−0.986***(0.143)	1.097***(0.184)
*lnPOP*	0.382***(0.066)	0.1773 (0.15)	0.382***(0.066)
*lnTOP*	0.470***(0.077)	0.0445 (0.044)	0.470***(0.077)
*DEMO*	−0.406*(0.21)	0.273*(0.14)	−0.406*(0.21)
Constant	−32.66***(1.729)	−4.023 (2.46)	−32.66***(1.729)
**Results of Hausman's (1978) specification test**			
Coefficient Value			
Chi-square test value 86.43			
p-value .000			
Prob > chi^2^ = 0.0000			

Notes: ***p < .01, **p < .05, *p < .1.

With the help of STATA software, the Hausman (1978) test results are also shown in Table 5 to decide whether a fixed effect model or a random effect model is better for the analysis. Here, the chi^2^ value is 86.43, and the corresponding probability is 0.000. The null hypothesis of random effect appropriateness is not accepted, which implies that the fixed effect model is the best model for our analysis. According to the FE findings, the estimated value of the *lnGDP* coefficient is 0.603; the rise in *CO*_*2*_ emissions would be 0.603% for every 1% growth in *GDP*. The predicted *lnGDP*^*2*^ coefficient has a negative but insignificant effect on carbon emissions. The result provides weak evidence for the EKC hypothesis.

Furthermore, the outcome of the fixed effect model demonstrates that the consumption of renewable energy (*lnREN*) is significant and negative at a 1% level of significance, which implies that a 0.986% reduction in carbon emissions results from every percentage increase in renewable energy consumption. Population growth's estimated coefficient value is positively insignificant. Additionally, trade openness is not a significant factor in explaining carbon emissions. Lastly, the outcomes of the *DEMO coefficient* show that democracy is positive and significant (0.273) with a 10% significance level. Every 1% increase in democracy (*DEMO*) leads to a rise in carbon emissions of 0.273%. Even practicing democracy cannot reduce carbon emissions if corruption continues to exist.

Before analyzing the results of pooled OLS regression, it is essential to check the multicollinearity among the independent variables. According to Ref. [97]; whether the VIF is equal to or above 2.5 is a matter of concern. However, the VIF result in Table 7 does not violate their finding.

**Table 7:** VIF test for independent variables.

Variable	VIF	1/VIF
*lnGDP*	1.70	0.588235
*lnGDP*^*2*^	1.09	0.917431
*lnREN*	1.46	0.684932
*lnPOP*	1.99	0.502512
*lnTOP*	1.88	0.531914
*DEMO*	1.29	0.775194
Mean VIF	1.57	

The study may evaluate the factors affecting carbon emissions across the conditional mean with the support of quantile regression. The results from pooled OLS regression, the results from fixed effects, and the results from various quantiles are all shown in Table 8, which helps to evaluate the outcomes quickly. In the case of *GDP*, the estimated coefficient has the anticipated positive sign and is significant in all quantiles with a 1% significance level. That indicates that *GDP* growth enhances *CO*_*2*_ emissions in these four selected South Asian countries. Our results from pooled, FE, and different quantiles demonstrate that carbon emissions are very responsive to changes in *GDP*. As for *GDP*^*2*^, pooled OLS, FEM, and quantile results negatively impact carbon emissions. The pooled OLS outcome is significant, with less than a 1% p-value. In the fixed effect model, the estimated coefficient value of *lnGDP*^*2*^ is insignificant. Quantile analyses reveal that *lnGDP*^*2*^ reduces *CO*_*2*_ emissions at all quantiles, but the significance varies from quantile to quantile. The estimate of *lnREN* exerts a negative and significant impact only in the fixed effect model. However, renewable energy consumption is significantly positive in the estimated pooled OLS and quantile regression approaches. The effect of *lnPOP*, which is significant at every quantile and in the pooled OLS regression, is another interesting finding. Environmental deterioration is accelerated by population growth, which is significant and exhibits the predicted positive trend across the distribution. *lnPOP* is not relevant just in terms of FEM's estimation. The quantile estimates in our analysis reveal a positive impact of trade openness on carbon emissions at all quantiles. Its significance varies across quantiles. From the pooled OLS regression results perspective, trade openness positively and significantly influences *CO*_*2*_ emissions. Democracies (*DEMO*) can significantly reduce carbon emissions at the 50th, 75th, and 95th quantiles. In contrast, the FEM analysis demonstrates that democracy significantly and positively influences carbon dioxide emissions.

**Table 8:** Results of simultaneous panel quantile regression [2,8,92]].

Variable *lnCO*_*2*_	OLS pooled	OLS Fixed	Simultaneous panel quantile regression estimator			
			Q_0.25_	Q_0.5_	Q_0.75_	Q_0.95_
*lnGDP*	1.213***	0.603***	1.181***	1.415***	1.125***	1.220***
*lnGDP*^*2*^	−0.216***	−0.0128	−0.255*	−0.207**	−0.065	−0.032
*lnREN*	1.097***	−0.986***	0.796**	1.449***	1.170***	1.2912***
*lnPOP*	0.382***	0.177	0.355***	0.283*	0.396***	0.321***
*lnTOP*	0.47***	0.044	0.474***	0.382*	0.172	0.103
*DEMO*	−0.406*	0.272*	−0.114	−0.993*	−0.625***	−0.900***
Constant	−32.66***	−4.02243	−30.425***	−36.84***	−29.842***	−30.951***
Obs	115	115	115	115	115	115

Notes: ***p < .01, **p < .05, *p < .1.

The study's key finding, as shown in Table 9, is the outcome of the innovative [57] quantile regression. We use the Powell quantile regression method with quantiles of 0.25, 0.50, 0.75, and 0.95 to describe the differential effects of *GDP*, *GDP*^*2*^, renewable energy consumption, population, trade openness, and democracy on carbon dioxide emissions in four selected South Asian countries. At all quantiles, the effect of *GDP* on *CO*_*2*_ emissions is positively significant, similar to the outcomes of OLS pooled and OLS fixed. Environmental quality suffers from the rising *GDP*. Industries are constantly increasing their production to boost economic growth. In this process, fossil fuels and non-eco-friendly technologies are increasing. As a result, the amount of carbon dioxide in the atmosphere is increasing daily. Several previous studies, including [[98], [99], [100]]; and [59]; support that per capita *GDP* has a positive impact on carbon dioxide emissions. However, this outcome is incompatible with the conclusions of [101]; whose research stated that the economy's growth aids in reducing ecological footprints [102]. also indicated that a 1% change in the increase of per capita *GDP* results in a 0.11% reduction in *CO*_*2*_ emissions.

**Table 9:** Results of [57] quantile regression approach.

Variable *lnCO*_*2*_	OLS pooled	OLS Fixed	[57] panel quantile regression estimator			
			Q_0.25_	Q_0.5_	Q_0.75_	Q_0.95_
*lnGDP*	1.213***	0.603***	1.685***	1.936***	1.536***	1.348***
*lnGDP*^*2*^	−0.216***	−0.012	−0.087***	−0.123***	−0.154***	−0.096***
*lnREN*	1.097***	−0.986***	0.555***	0.496	1.068***	1.137***
*lnPOP*	0.382***	0.177	0.023	−0.238	0.196*	0.306***
*lnTOP*	0.47***	0.044	0.345***	−0.058	0.208*	0.360***
*DEMO*	−0.406*	0.272*	−0.670***	−1.244***	−1.11***	−0.794***
Obs	115	115	115	115	115	115

Notes: ***p < .01, **p < .05, *p < .1.

All quantiles show a negative impact of *GDP*^*2*^ on carbon dioxide emissions, which is significant in all quantiles according to statistical analysis. Therefore, the positive sign of the estimated *lnGDP* and the negative sign of the estimated *lnGDP*^*2*^ indicate a reversed U-shaped association between economic growth and carbon emissions, confirming the EKC hypothesis's validity for the selected South Asian countries. Carbon emissions will decrease if the countries invest in clean and sophisticated technologies to increase production while reducing pollution. The findings are similar to a variety of past studies, consisting of [22,24]; and [103]. Several studies contradict our findings, consisting of [104] for the nations of the OECD [63], for Asian economies, and [70] for West Africa.

Our findings from the quantile estimates examine the association between the consumption of renewable energy and carbon dioxide emissions, where its significance differs between quantiles. The majority of prior research indicates that renewable energy consumption and *CO*_*2*_ emissions are negatively and significantly related [27,[105], [106], [107], [108]]. Wind and solar power sources can create energy without emitting carbon dioxide, but their production and transportation still produce carbon emissions. Also, biomass is an essential renewable energy source that damages the atmosphere the most [29]. Environmental pollution from burning wood and other solid waste households use to prepare food can be severe. At the 25th, 75th, and 95th quantiles, consumption of renewable energy affects *CO*_*2*_ emissions not only positively but also significantly. The positively significant association between renewable energy consumption and environmental degradation is similar to previous studies, including [65,109].

Population growth affects *CO*_*2*_ emissions depending on the quantile, as shown by Ref. [57] quantile regression results, which vary across quantiles. A positively significant correlation between the growth of the population and *CO*_*2*_ emissions is found at the 75th and 95th quantiles [110]. provided evidence supporting the conclusions and showed how population density increases carbon dioxide emissions in the atmosphere. The fundamental cause of all current environmental degradation issues worldwide is population expansion. A wide variety of research done in the past, consisting of [31,32,111]; and [59]; reported these outcomes. Population growth considerably strains the world's energy sources, land, water, air, and forests, deteriorating the environment and accelerating worldwide warming and climate change. This result is dissimilar to that of [33,61].

The calculated values of [57] quantile regression approach at the 25th, 75th, and 95th quantiles indicated that trade openness (*lnTOP*) has a significant as well as positive influence on *CO*_*2*_ emissions, while the estimated quantiles of *lnTOP* are insignificantly negative at the 50th quantile. Trade openness increased a nation's exports, raising economic output by expanding the size of industries. This process simultaneously raises pollution levels in the nation [112]. This positive and significant influence is also justified by Refs. [70,113]; and [114]. However, the finding contradicts [38] and Shekhawat et al. (2021).

According to our findings, the influence of democracy (*DEMO*) on *CO*_*2*_ emission values may differ between quantiles. All of the quantiles, including the 25th, 50th, 75th, and 95th, indicate that the effect of democracy on *CO*_*2*_ emissions is negatively significant. This finding is consistent with previous research by Ref. [43] for 19 emerging economies; Adams et al. (2019) for Sub-Saharan Africa [115]; for BRICS nations; Jahangir et al. (2021) for selected developing nations [116]; for France. Democracies appear more effective when they demonstrate deeper commitments to lowering carbon dioxide emissions by enacting various tax systems, laws, and regulations. In selected South Asian nations, democratic systems assist citizens in making more ecologically responsible decisions to lessen ecological problems. [73,117]; and Midlarasky (1998) all came to different conclusions, which do not match the results.

In Table 10, the Wald test results from our analysis show that the estimated slope coefficients for each quantile are statistically significant with a 1% significance level, which suggests that different variables at different quantiles have different effects on *CO*_*2*_ emissions.

**Table 10:** Results of the Wald test.

Variable	The levels of quantiles			
	0.25	0.50	0.75	0.95
lnGDP – lnGDP*	5005.3***	2128.3***	10063***	38324***
*lnGDP*^*2*^*– lnGDP*^2^*	3658.7***	1399.1***	5839***	36738***
lnREN –lnREN*	3085.4***	123.4***	10440***	33378***
lnPOP –lnPOP*	32470.8***	3505.1***	34875***	308405***
lnTOP –lnTOP*	1130.6***	260.2***	10077***	25734***
DEMO–DEMO*	2291.2***	1106.8***	3071.5***	12618***

Notes; ***p < .01, **p < .05, *p < .1.

Table 11 displays the remarkable findings obtained from the panel causality test conducted by Ref. [60]. The null hypothesis is not accepted with a 1% significance level, which leads us to conclude that a two-way causality exists between carbon dioxide emissions and *GDP*. However, this investigation did not reveal any causal association between *lnGDP* and *lnCO*_*2*_, which suggests unidirectional causality. The outcomes from the D-H causality test demonstrate that there is one-way or unidirectional causality between *lnCO*_*2*_ and *lnREN* or *lnREN* and *lnCO*_*2*_. The bidirectional or two-way causalities run from carbon emissions to population growth and from population growth to carbon emissions. A bidirectional causal relationship also exists between *lnCO*_*2*_ and *lnTOP*. The D-H outcomes of *lnTOP* to *lnCO*_*2*_ imply that there exists an insignificant link between trade openness and *CO*_*2*_ emissions. Finally, the D-H causality test outcome for *lnCO*_*2*_ to *DEMO* suggests a unidirectional causality between carbon emissions and democracy. Furthermore, this research identifies bidirectional causality between *DEMO* and *lnCO*_*2*_.

**Table 11:** Results of the D-H (2012) panel causality test.

Null hypothesis (H_o_)	Z-bar	Z-bar tilde	Decision
*lnCO*_*2*_*≠lnGDP*	3.44***	2.87***	Bidirectional
*lnGDP≠lnCO*_*2*_	−0.46	−0.50	Unidirectional
*lnCO*_*2*_*≠lnGDP*^*2*^	–	–	–
*lnGDP*^*2*^*≠lnCO*_*2*_	–	–	–
*lnCO*_*2*_*≠lnREN*	0.65	0.46	Unidirectional
*lnREN≠ lnCO*_*2*_	1.00	0.76	Unidirectional
*lnCO*_*2*_*≠ lnPOP*	5.83***	4.95***	Bidirectional
*lnPOP≠ lnCO*_*2*_	63.73***	55.08***	Bidirectional
*lnCO*_*2*_*≠lnTOP*	2.97***	2.47**	Bidirectional
*lnTOP≠ lnCO*_*2*_	−0.10	−0.19	Unidirectional
*lnCO*_*2*_*≠DEMO*	0.89	0.66	Unidirectional
*DEMO≠ lnCO*_*2*_	6.83***	5.81***	Bidirectional

Notes; ***p < .01, **p < .05, *p < .1.

## 5 Discussion

Earth's temperature is regrettably rising. The conscious community has recently become aware of the seriousness of global warming because it has catastrophic effects on the environment. People's health, agricultural production, quality of life, and the environment are at risk [118]. Coal and diesel are the two most important forms of fossil fuel, and the global use of these substances is at an all-time high. Because of this, the amount of *CO*_*2*_ released into the atmosphere has significantly grown. Technology friendly to the environment is a workable plan for addressing environmental problems and achieving carbon neutrality. However, it may also assist governments in pursuing noticeable economic growth [119].

First, our estimated findings suggest that *GDP* growth is positively associated with carbon emissions in four selected South Asian countries. An essential factor in the economic growth of most countries, including developed and developing economies, is the heavy reliance on non-renewable energy sources to meet their energy needs, which worsens environmental quality [120,121]. When the four South Asian countries reach a certain income level, they start using ecologically friendly green technology in their production, which helps to reduce carbon emissions gradually. Our research findings also suggest that the reversed “U"-shaped (EKC) curve is validated for these four countries. Moreover, the rise in squared *GDP* improves the environmental standards in South Asia [27,121,122]. According to our research analysis, renewable energy consumption is positively associated with carbon dioxide emissions. In South Asian nations, underdeveloped rural areas depend on air-polluting biomass, biogas, and biofuels. They burn solid waste and wood in food production, which is detrimental to the environment. These selected South Asian nations use less geothermal, wind, and hydropower energy. In electricity production, these nations rely more on fossil fuels such as coal, natural gas, and crude oil than renewable energy. The overuse of biomass and biofuels negatively impacts environmental sustainability, which was confirmed by the research of [123]. Due to the increase in population, housing and infrastructure must be built for the additional people, resulting in urbanization and industrialization, which are responsible for the destruction of forests and land degradation. As a result, the ecological balance is destroyed. Chemical fertilizers and pesticides are being used on the land to grow more food grains and crops to meet the food needs of the growing population. As a result, soil fertility is being lost, and water is being polluted. Vehicle exhaust pollutes the atmosphere, and biodiversity is threatened, disrupting the food chain. Thus, population growth worsens ecological conditions. Our research indicates that *CO2* emissions in four selected South Asian countries positively correlate with population growth. Population growth is known to be one of the key factors contributing to the degradation of the environment in the countries of South Asia [124,125]. *The people of South Asia need to be more active and aware of the importance of adopting environmentally friendly technologies since they do not participate in environmental education programs.* The process of globalization has increased economic growth in the world economy. The growth trend is related to the environment. Trade openness mainly helps to reduce production costs. This trade openness establishes more industrial units and structures to increase production [126,127]. Trade openness increases the exporting country's domestic production, creating more fossil fuel demand and resource consumption, which degrade environmental quality [128]. Our investigation found that trade openness has significantly increased *CO2* emissions in these four selected South Asian countries. Most of the research pointed out that increased trade openness is positively associated with degradation in the quality of the natural environment [129]; Popexcu, 2016). Democracy plays a significant positive role in enhancing ecological conditions. Democracy should influence environmental quality positively for several reasons. Citizens of democracy are better informed about environmental issues and circumstances because of the greater availability of information.

Additionally, it promotes public participation in decision-making, which may help ensure that environmental goals are conveyed to those making the decisions. Under a democratic system, individuals can practice their choices for improving ecological quality more effectively than in an autocratic government. Because of this, the amount of pollution released goes down [[130], [131]].

## 6 The conclusion

The research attempts to assess the influence of *GDP* (*lnGDP*), *GDP2* (*lnGP2*), renewable energy consumption (*lnREN*), population (*lnPOP*), trade openness (*lnTOP*), and democracy (*DEMO*) on *CO2* emissions (*lnCO2*) in four selected countries of South Asia between the years 1990 and 2019. In our empirical research, we first apply the unit root tests—not only IPS and ADF but also CIPS and CADF—which confirm the variable's stationarity. In the second step of our process, we examine the findings of a cointegration test, which assure that variables are cointegrated in the long run. Thirdly, the research employed a pooled OLS regression approach, highlighting that *GDP*, renewable energy consumption, population growth, and trade openness are significantly responsible for carbon dioxide emissions. While squared *GDP* and democracy significantly decline carbon dioxide emissions rates and upgrade environmental quality. The estimate of *GDP* and *GDP2* confirms the validity of the reversed U-shaped curve (EKC). Fourthly, we discuss the results of the fixed effect and the random effect regression. The results of the Hausman test indicate that fixed-effect regression is appropriate for this research. According to FEM projections, renewable energy consumption has a negative impact on carbon emissions, whereas democracy has a positive effect on *CO2* emissions. Our study applied [57] innovative quantile regression method, which demonstrates varied consequences across quantiles and solves the problem of fixed effect regression. Results from Ref. [57] quantile regression show that the value of the GDP coefficient (1.685, 1.936, 1.536, and 1.348) is positive and significant at every quantile. The coefficient value of the *lnGDP2* (−0.087, −0.123, −0.154, and −0.096) is significantly negative at all quantiles. Positive and negative values confirmed a reversed U-pattern association between economic growth and environmental destruction. Variable renewable energy consumption (*lnREN*) is negatively and significantly associated with carbon emissions at the 25th, 75th, and 95th quantiles. The coefficient values of *lnPOP* (0.196 and 0.306) are positively and significantly associated with carbon emissions at the 75th and 95th quantiles. Also, trade openness positively correlates with carbon emissions at the 25th, 75th, and 95th quantiles.

Finally, at all quantile levels, the coefficient values of democracy (−0.670, −1.244, −1.11, and −0.794) reveal a significantly negative impact on *CO2* emissions. Based on the research findings, it can be said that through proper democratic practices, economic development and environmental protection can be continued simultaneously, which helps in the formation of EKC. According to the findings of the Wald test, each of the variables has an asymmetrical influence on the carbon dioxide emissions at each of the quantiles. The D-H panel causality test confirms two-way causality between variables such as *CO2* emissions and *GDP*, *CO2* emissions and population growth, population growth and *CO2* emissions, *CO2* emissions and trade openness, and democracy and *CO2* emissions. The main finding of this study is that the two-way causality shows a significant link, which suggests that policymakers should pay attention to these variables.

## 7 Policy recommendations

(1) A country's population is considered its greatest asset, but overpopulation burdens that nation. The main problem in developing countries is rapid population growth. Hence, the high population growth in these selected South Asian countries must be controlled. The only way to make such a state self-sufficient is to follow the population program fully. Policymakers work to implement various policies, such as a one-to-two-child policy that should be implemented to slow population growth; education campaigns on the value of birth control; encouragement of women's education; and campaigns to stop child marriage and polygamy. The government should take several actions to develop population policy, implement different initiatives for population development, arrange various programs for education, information, and research, and open a family welfare center.
(2) Energy consumption has increased along with economic expansion in the modern world, which has already had a significant negative impact on the environment. South Asian countries should vigorously implement energy-efficient technologies across the industry to cut carbon emissions. They should promote sustainable farming practices that lower carbon emissions. The best way to protect the planet's ecosystem is to encourage a recycling system in the manufacturing process. The countries should promote the adoption of solar energy and support green economies to reduce their dependency on fossil fuels.
(3) The government should use tax incentives to promote the sale of low-carbon goods while discouraging the sale of high-carbon goods to lower *CO*_*2*_ emissions [37]. The stakeholders should implement effective laws and policies that contribute to developing a sustainable world. The International Trade Association recommends that developing countries invest in sophisticated technologies and adopt low-carbon practices, such as producing eco-friendly goods, to reduce carbon footprints.
(4) Reducing the dependency of our selected South Asian countries on biomass is necessary because air pollution is a significant drawback of biomass, which has a harmful influence on the environment. The government should enact to use renewable energy in manufacturing within a predetermined timeframe. Exploration for low-carbon emission sources is urgently needed to reduce carbon emissions and environmental devastation. It is necessary to use renewable technology to achieve the goal of carbon neutrality. Water, wind, and solar energy can be used to generate electricity, which is efficient for the environment. The production cost of renewable energy is higher than other methods, although it is profitable in the long run. For this reason, investment in this sector should be increased.
(5) Our empirical analysis suggests that democracy should be prioritized to reduce carbon emissions. The selected South Asian nations should fortify their democracies to safeguard the environment. The democratic state system should practice a healthy form of democracy. Additionally, improved democratic accountability will strengthen environmental laws and expand low-carbon energy consumption. South Asian countries must strengthen intergovernmental cooperation to cut carbon emissions and meet the goals of COP-27. Deforestation increases worldwide as a result of human activities like urbanization and industrialization. A democratic government can take various measures to prevent the cutting of trees. Moreover, different types of tree plantation programs can be undertaken. The stakeholders can encourage people to use energy-efficient technology and renewable energy.

## 8 Limitations and scope of the future study

Since the study only covers the data period from 1990 to 2019, the research does not capture the picture of the deterioration of the state of democracy in South Asia during 2020–23. In this study, the data on renewable energy consumption mainly shows the total final energy consumption percentage, whereas the data does not refer to renewable energy sources. Due to missing data, the study is based on four selected South Asian countries, which fails to show the complete picture of South Asia. This study only shows the impact of one region. In the future, the study should focus on other least-developed, developing, and developed economies; more effective policies can be found by comparing them. The study examined only CO_2_ emissions. However, future research should expand using the ecological footprint (EF), which has recently become more popular as a broad environmental quality measure.

## Author contribution statement

Tasnim Sultana: Conceived and designed the experiments; Contributed reagents, materials, analysis tools or data; Wrote the paper.

Md. Shaddam Hossain: Performed the experiments; Analyzed and interpreted the data.

Liton Chandra Voumik: Asif Raihan: Performed the experiments; Wrote the paper.

## Data availability statement

Data will be made available on request.

## Funding statement

This research received funding from Research Cell, Noakhali Science and Technology University

## Consent for publication

N/A.

## Declaration of competing interest

The authors declare that they have no known competing financial interests or personal relationships that could have appeared to influence the work reported in this paper.
